# Genome-Wide Analysis of Cell Cycle-Regulating Genes in the Symbiotic Dinoflagellate *Breviolum minutum*

**DOI:** 10.1534/g3.119.400363

**Published:** 2019-09-24

**Authors:** Michael L. Cato, Hallie D. Jester, Adam Lavertu, Audrey Lyman, Lacey M. Tallent, Geoffrey C. Mitchell

**Affiliations:** * Department of Biology, Wofford College, Spartanburg, SC, 29303 and; † Department of Biology, Colby College, Waterville, ME, 04901

**Keywords:** cell cycle, cyclins, cyclin-dependent kinases, dinoflagellate, Symbiodiniaceae, *Breviolum*, *Symbiodinium*, *Aiptasia*

## Abstract

A delicate relationship exists between reef-building corals and their photosynthetic endosymbionts. Unfortunately, this relationship can be disrupted, with corals expelling these algae when temperatures rise even marginally above the average summer maximum. Interestingly, several studies indicate that failure of corals to regulate symbiont cell divisions at high temperatures may underlie this disruption; increased proliferation of symbionts may stress host cells by over-production of reactive oxygen species or by disrupting the flow of nutrients. This needs to be further investigated, so to begin deciphering the molecular mechanisms controlling the cell cycle in these organisms, we used a computational approach to identify putative cell cycle-regulating genes in the genome of the dinoflagellate *Breviolum minutum*. This species is important as an endosymbiont of *Aiptasia pallida*—an anemone that is used as a model for studying coral biology. We then correlated expression of these putative cell cycle genes with cell cycle phase in diurnally growing *B. minutum* in culture. This approach allowed us to identify a cyclin/cyclin-dependent kinase pair that may function in the G1/S transition—a likely point for coral cells to exert control over algal cell divisions.

A critical relationship exists between reef-building corals and the symbiotic algae residing within them. These dinoflagellates (colloquially, zooxanthellae), from the family Symbiodiniaceae (recently revised taxonomically (LaJeunesse *et al.* 2018)), are photosynthetic, harvesting energy from sunlight and sharing that energy with their coral hosts. In return, corals provide them with a source of nitrogen, a stable position in the water column, and protection from grazing. This relationship, as important as it is for reef health, is delicate—exposure to temperatures only marginally above the average summer maximum can cause corals to bleach, expelling their resident algae (Hoegh-Guldberg *et al.* 2007; Hughes *et al.* 2017). Massive bleaching due to global warming will drastically and irreversibly alter coral reef ecosystems around the world, adversely impacting fisheries, coastal ecosystems, and placing financial strain on developing economies that depend on tourism (Hoegh-Guldberg *et al.* 2017; Hughes *et al.* 2018).

Remarkably, studies suggest that failure of the host to properly regulate symbiont cell divisions at high temperatures may cause bleaching (Bhagooli and Hidaka 2002; Strychar *et al.* 2005). It is possible that increased rates of algal division stress host cells through over-production of reactive oxygen species or by disrupting the exchange of nutrients. In some corals, the optimal growth temperature for the symbiont may, in fact, exceed the bleaching threshold. For example, Symbiodiniaceae (*Cladocopium* sp.) isolated from *Montipora capitata* proliferate better when reared at 31° than when reared at lower temperatures (Kinzie *et al.* 2001); this temperature, however, is lethal to the host (Jokiel and Coles 1977).

To establish symbiosis, some corals seem to produce a chemical signal that forces symbionts into a non-motile, dividing state (Koike *et al.* 2004). Once in that state, it is unclear how algal cell divisions are coordinated by corals to ensure that the proper density of symbionts is maintained. Several pre-mitotic mechanisms have been proposed in a variety of Cnidarians, including factors produced by coral cells that inhibit the algal cell cycle (Smith and Muscatine 1999) and limited access to nutrients (Falkowski *et al.* 1993), which may be controlled by the host or simply by competition among symbionts (consistent with (McAuley and Darrah 1990)). Post-mitotic regulation might include digestion of algae *in situ* (Titlyanov *et al.* 1996) or expulsion of excess algae (Jones and Yellowlees 1997). In the anemone *Aiptasia pallida* (formally *Exaiptasia pallida*; synonymous with *Aiptasia pulchella* (Grajales and Rodríguez 2014; Grajales and Rodríguez 2016)), rates of algal expulsion increase with temperature (Bieri *et al.* 2016) and expelled algae, surprisingly, have a much higher rate of mitosis than algae that are retained within their hosts (Baghdasarian and Muscatine 2000). Regardless of the mechanism(s) responsible for maintaining a healthy symbiont density, it is evident that controlling algal cell divisions *in hospite* is important.

The eukaryotic cell cycle is divided into four distinct phases—two gap phases (G1 and G2) interrupted by DNA replication (synthesis/S phase) and followed by mitosis (M), which ends with cytokinesis. Moving successively from one stage to another is governed through phosphorylation of substrates by the cyclin-dependent kinases (CDKs). As the name implies, these proteins are active only when paired with a partner cyclin that can target them to the appropriate substrates. The CDKs can be additionally activated by CDK-activating kinases (CAKs) (Kaldis 1999) and Cdc25 phosphatases and inhibited by members of the WEE1/MYT1 kinase family (Perry and Kornbluth 2007) . Further, the CDK-interacting protein CKS1 is essential for both the G1/S and G2/M transitions in some organisms (Tang and Reed 1993). Meanwhile, the cyclin-dependent kinase inhibitors (CKIs)—*e.g.* the mammalian proteins p21^Cip1/Waf1^ and p27^Kip1^—can initiate cell cycle arrest in response to internal or external stimuli (El-Deiry *et al.* 1994; Polyak *et al.* 1994).

While checkpoints regulate each cell cycle transition, eukaryotic cells commit to division during G1. In yeast, this point of commitment is called START (Hartwell *et al.* 1974), while in metazoans it is referred to as the restriction point (R-point) (Pardee 1974). In general, the cell cycle is only sensitive to external factors prior to this; once a cell has committed to divide, it can do so without any further input. In light of this, if a Cnidarian host is regulating the proliferation of its symbionts, it makes sense that it would do so by blocking the R-point. In fact, symbiotic algae in *Aiptasia pallida* spend considerably longer in G1 than the same species of algae (*B. minutum*) in culture, despite the durations of S, G2, and M remaining relatively consistent (Smith and Muscatine 1999).

As a first step toward our goal of understanding how coral cells regulate the divisions of their symbiotic algae, we identified key cell cycle regulators in the genome of *Breviolum minutum*. While the family Symbiodiniaceae is quite diverse (as evidenced by its recent reclassification (LaJeunesse *et al.* 2018)), we chose this species because it is an important endosymbiont in the sea anemone *Aiptasia pallida*, which is becoming a common model organism for studying many aspects of coral biology (Santos *et al.* 2002; Weis *et al.* 2008; Baumgarten *et al.* 2015). We then correlated the expression of those genes with cell cycle phase in diurnally growing *B. minutum* in culture. This approach allowed us to find specific cyclins and CDKs that may be involved in the G1/S transition—a likely point for coral cells to exert control over algal cell divisions.

## Materials and Methods

### Identification of putative cell cycle-regulating genes

Amino acid sequences for all annotated CDKs in *H. sapiens*, *A. thaliana*, and *S. cerevisiae* were downloaded from GenBank. A multiple sequence alignment constructed using the Probcons algorithm with default parameters (Do *et al.* 2005), was used to generate a CDK-specific hidden Markov model (HMMER 3.1b1, hmmer.org). This model was constructed from such diverse taxa to allow identification of a large assortment of novel CDK-related genes in *B. minutum*. To do this, HMMER was used to query our hidden Markov model against the predicted proteome of *B. minutum* established by Shoguchi *et al.* (2013) and made available through the Okinawa Institute of Science and Technology (marinegenomics.oist.jp/symb/viewer/info?project_id=21). Initial results were culled by removing all high scoring pairs (HSPs) with fewer than 200 residues and any HSPs that didn’t begin within 55 bases of the start of the CDK alignment. To identify novel cyclins, Cdc25 phophatases, MAT1 homologs, and CKS1 homologs in *B. minutum*, hidden Markov models were retrieved from PFAM (pfam.xfam.org) and queried against the predicted proteome of *B. minutum*, again using HMMER. For the cyclins, a PFAM model describing the characteristic N-terminal domain that is common to all cyclins was chosen. All potential CDK, cyclin, Cdc25, MAT1, and CKS1 sequences were then reciprocally queried against the UniProtKB database using the HMMER web server (Finn *et al.* 2011). Those sequences that had CDKs, cyclins, Cdc25 phophatases, MAT1, or CKS1 as top hits were used for further analysis unless they carried additional domains that made them likely members of another protein family (*e.g.*, an N-terminal cyclin domain in a putative CDK).

### Gene model refinement

In cases where we initially failed to detect a predicted transcript (see Quantitative RT-PCR below) or had another cryptic result, we used the BLAST server available at reefgenomics.org to query these sequences against available *Breviolum* transcriptomes (*B. minutum*, *B. pseudominutum*, *B. aenigmaticum*, *B. psygmophilum*) (Parkinson *et al.* 2016). In several cases, what was defined as a gene by Shoguchi *et al.* (2013) coded for multiple transcripts (*e.g.*, Figure S1). We identified the transcript of interest by finding the open reading frame identified as a CDK-related sequence, cyclin, MAT1, or CKS1 homolog. The cDNA sequences used for subsequent analyses are available in File S1, while File S2 contains the peptide sequences.

### Phylogenetic analysis

MUSCLE (Goujon *et al.* 2010) and Probcons (Do *et al.* 2005) were used with default parameters to create peptide alignments of putative *B. minutum* CDKs with representative CDKs from *H. sapiens* and two well-characterized Apicomplexans—*Plasmodium falciparum* (sequences retrieved from PlasmoDB, plasmodb.org), and *Toxoplasma gondii* (sequences retrieved from ToxoDB, toxodb.org). The Apicomplexans share the superphylum Alveolata with Symbiodiniaceae, and thus are ideal for this analysis. Gaps were removed from the alignment before using MEGA X (Kumar *et al.* 2018) to generate neighbor joining and maximum likelihood trees with 1000 bootstraps (for further details, see specific figure legends). For cyclins, alignments of the N-terminal domains of the novel *B. minutum* sequences and representative cyclins from *H. sapiens*, *P. falciparum*, and *T. gondii* were created using MUSCLE and Probcons with default parameters for peptide alignment. Gaps were removed from the alignment and trees were constructed as they were for the CDKs.

### Cell culture

Cultures of *B. minutum* were acquired from the National Center for Marine Algae and Microbiota (Boothbay Harbor, ME; accession number CCMP3345). They were grown in flasks containing f/2 (National Center for Marine Algae and Microbiota, Boothbay Harbor, ME) made from filtered sea water collected from the Gulf of Maine supplemented with 50 mg/ml each of kanamycin, ampicillin, and streptomycin, but without further addition of silica. A period of 13 h of light (approximately 100 μmol*photons*m^-2^*s^-1^) followed by 11 h of darkness was maintained in an environmental growth chamber set to 25°. Experiments were performed on cultures undergoing log-phase expansion (1-2 × 10^6^ cells/ml).

### Flow cytometry

To determine the fraction of cultured cells in each cell cycle phase across a 24 h day-night cycle, 1 × 10^6^ cells were collected. They were centrifuged for 5 min at 200 × g, fixed in 1 ml of 70% ethanol for 1 h on the bench, washed with PBS, and resuspended in 500 μl of staining buffer (PBS, 0.1% Triton X-100, 20 μg/ml propidium iodide (Sigma-Aldrich), and 10 μg/ml RNase A). Cells stained with propidium iodide were run through a FACSCalibur (BD Biosciences, San Jose, CA) set to detect 585 nm fluorescence upon excitation at 488 nm. Data were collected for 10,000 cells and analyzed using FlowJo v.10.1 (Ashland, OR).

### Quantitative RT-PCR

To measure the expression of putative cell cycle regulators in cultured cells across a 24 h day-night cycle, 2 × 10^7^ cells were collected by centrifuging at 5,500 × g for 15 min, media was removed, and pellets were flash frozen in liquid nitrogen. RNA extraction combined lysis and homogenization in TRIzol (Life Technologies, Carlsbad, CA) with a kit-based cleanup following the protocol established by Rosic and Hoegh-Guldberg (2010). Briefly, pellets were resuspended in 1 mL of TRIzol, mixed thoroughly by pipetting, and transferred to screw-cap tubes with 0.3 g of glass beads (Sigma-Aldrich, St. Louis, MO). Cells were then disrupted twice for 90 sec in a MagNA Lyser (Roche Life Science, Basel, Switzerland) at 4,500 rpm. Debris was removed by centrifuging at 12,000 × g for 1 min, and supernatant was run through the RNeasy Plus Mini Kit following manufacturer’s instructions (Qiagen, Hilden, Germany). RNA was eluted in 30 μl of nuclease-free water and quantified on a NanoDrop Lite (Thermo Fisher Scientific, Waltham, MA). 1 μg of RNA was reverse transcribed using a QuantiTect Reverse Transcription Kit (Qiagen) following manufacturer’s instructions; this kit includes a genomic DNA wipeout step. cDNA was diluted 1:5 in nuclease-free water before being used for quantitative PCR. Quantitative PCR reactions were setup as follows: 10 μl QuantiNova SYBR Green PCR Master Mix (Qiagen), 1.5 μl mixed forward and reverse primer (10 μM each, Table S1), 4.5 μl of nuclease-free water, and 4 μl of template. Reactions were run on a CFX96 (Bio-Rad, Hercules, CA) at 95° for 2 min for initial activation following by 40 cycles of 95° for 5 sec then 60° for 10 sec. Data were normalized to the expression of S-adenosyl methionine synthase (SAM) (Rosic *et al.* 2011) and analyzed using the 2^-ΔΔCt^ method (Livak and Schmittgen 2001). Outliers, indicated by Grubbs’ test, were removed and statistical significance was determined using one-way ANOVA with a Dunnett’s multiple comparisons test in Graphpad Prism 8 (San Diego, CA).

### Data availability

**Figure 1:**
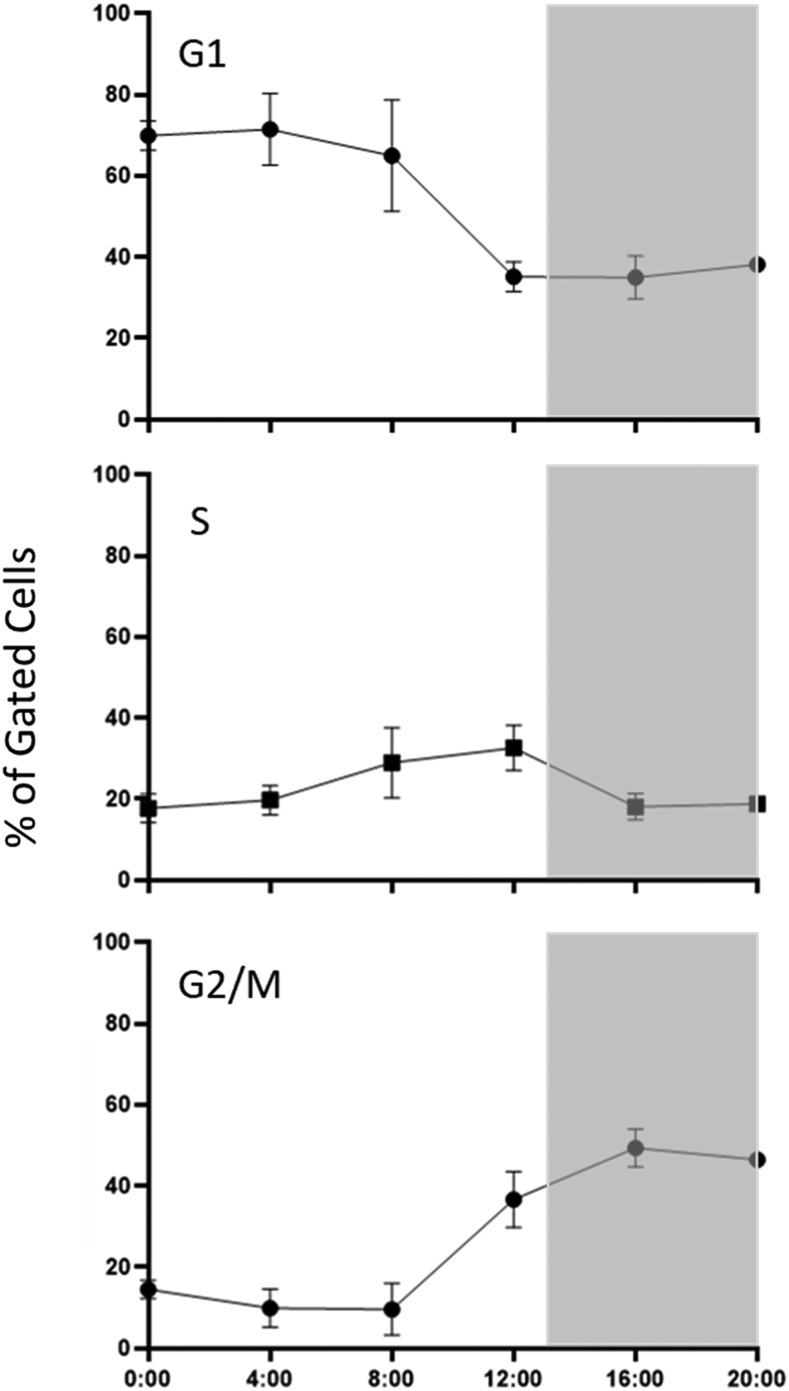
Cell cycle distribution of free-living *B. minutum* over 24 h. Four cultures of *B. minutum* maintained on a diurnal cycle were sampled every 4 h. Cells were stained with propidium iodide and nuclear content was assessed by flow cytometry. Data were analyzed using the FlowJo Cell Cycle platform and plotted as the average percentage of gated cells in each cell cycle phase ± SD. Gray background represents periods of darkness.

**Figure 2:**
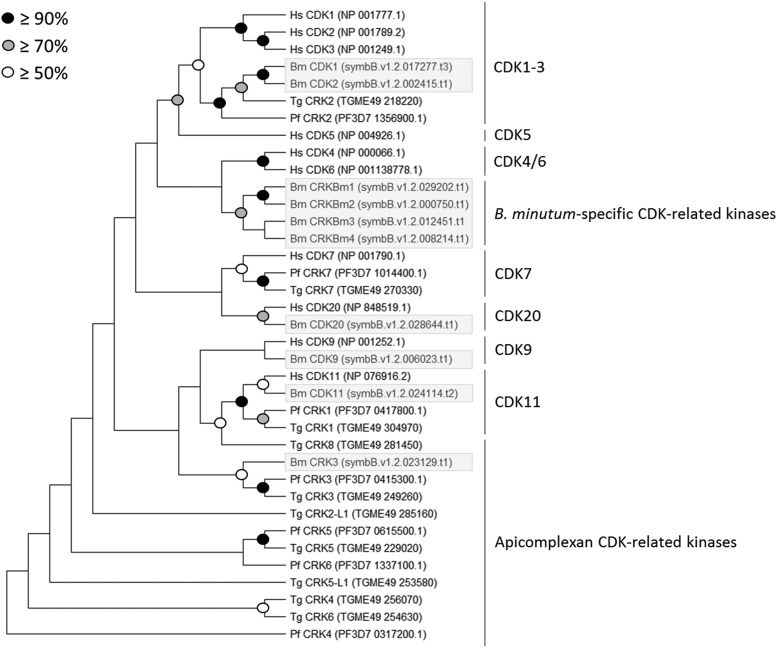
Phylogenetic analysis of putative CDKs and CDK-related kinases in *B. minutum*. Protein sequences for 10 CDKs and CDK-related kinases identified in the genome of *B. minutum* were aligned with representative CDK and CDK-related sequences from other species. Using MEGA X, gaps were removed and phylogenetic trees were created. This tree was constructed from a MUSCLE alignment using neighbor joining with 1,000 replicates. Accession numbers or specific genome identifiers (for *B. minutum*) are indicated next to the sequence ID.

File S1 contains cDNA sequences for all putative CDKs, cyclins, and CDK/cyclin interacting partners identified in this study. File S2 contains peptide sequences for all putative CDKs, cyclins, and CDK/cyclin interacting partners identified in this study. File S3 contains example flow cytometry profiles used for cell cycle analysis. File S4 contains raw flow cytometry data (FCS files) for Figure 1; individual FCS files are ordered by time-point (three files for each timepoint from 0:00 to 20:00). File S5 is a MUSCLE alignment of all CDK sequences used to generate the phylogeny in Figure 2. File S6 is a MUSCLE alignment of all cyclin sequences used to generate the phylogeny in Figure 6. File S7 is an Excel document containing all quantitative RT-PCR analyses with a separate tab for each gene. Supplemental material available at FigShare: https://doi.org/10.25387/g3.9808433.

## Results and Discussion

Division of free-living Symbiodiniaceae is regulated in response to light, so cells grown on a diurnal cycle (13 h of light and 11 h of darkness) were sampled every 4 h over one 24 h period to determine the percentage of cells in each cell cycle phase at a given time. Cells were stained with the nuclear stain propidium iodide, and nuclear content was assessed by flow cytometry. The highest percentage of G1 cells—about 70%—was observed at the onset of the light period (time 00:00) and persisted for 8 to 12 h (Figure 1). S phase peaked just before the initiation of the dark period (12:00), with the largest percentage of G2/M cells and lowest percentage of G1 cells observed a few hours later (16:00). These data imply that cytokinesis occurred in the hours immediately prior to the start of the light period. These results are consistent with published data, which show that the vast majority of free-living Symbiodiniaceae are in G1 early in the day and that the proportion of G2/M cells peaks during the dark period (Smith and Muscatine 1999; Wang *et al.* 2013). Interestingly, our results indicate a background presence of S phase cells (about 10%) at all time points. While this result is difficult to explain, the peak of roughly 30% S phase cells at 12:00 is consistent with the percentage of cells entering G2/M between 08:00 and 16:00. Similarly, by comparing the percentage of G2/M cells at 20:00 (46%) with the percentage of G2/M cells at 00:00 (15%), it is apparent that about 30% of the population divides overnight. This result seems reasonable in light of the 2.2 day doubling time of cultured *B. minutum* undergoing log-phase expansion (Figure S2).

When attempting to assign molecular events, such as changes in gene expression, to a particular cell cycle phase, it is commonplace to use a synchronized cell population; this is often achieved by treating with a mitosis inhibitor such as nocodazole. Although this approach has advantages, we chose to use a partially synchronized population (*i.e.*, only about 70% of cells were in G1 at time 00:00). First of all, cell cycle inhibitors are often cytotoxic, and nocodazole specifically is known to affect gene expression (Cho *et al.* 2006). Second, while darkness leads to the accumulation of cultured *Breviolum* cells in G1, we were unable to increase the percentage of G1 cells with prolonged darkness (Figure S3). Still, with roughly 30% of cells entering S phase by 12:00, we might expect to see increased expression of some genes involved in the G1/S transition at that time. Likewise, the proportion of G2/M cells drops precipitously (again, by about 30%) within the last 4 h of darkness, so we might expect to see increased expression of some genes involved in mitosis during this period.

### CDKs

**Figure 3:**
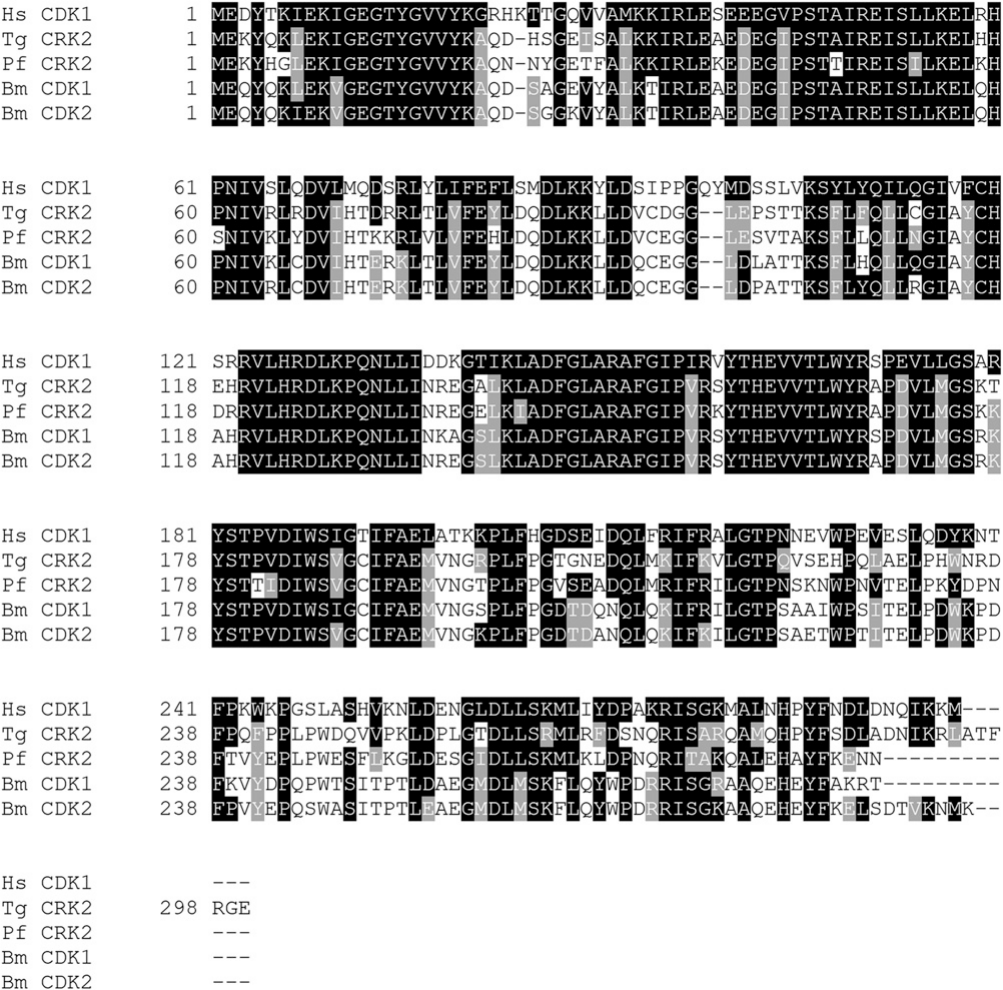
Alignment of the highly conserved CDK1-3 family. Protein sequences for selected CDK1-3 family members from other species were aligned to the putative *B. minutum* CDK1 using MUSCLE with default parameters. The BoxShade web server (expasy.org/resources) was used to shade the results of the alignment. Black boxes indicate an identical match to the consensus sequence (Hs CDK1). Gray boxes indicate residues that are biochemically similar to the consensus sequence.

**Figure 4:**
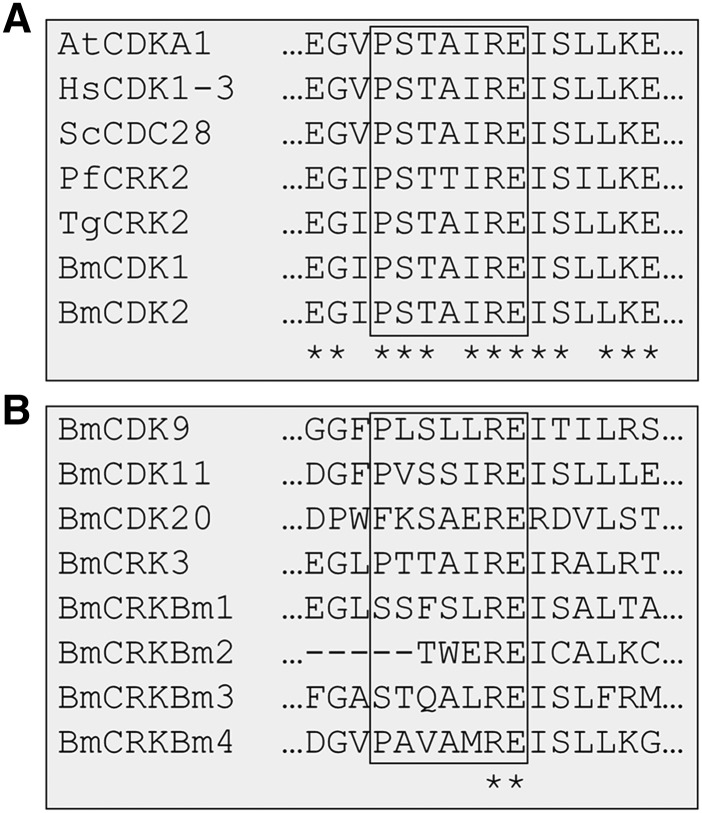
The cyclin binding motif in the CDK family. Using MUSCLE with default parameters, protein sequences for CDK1-3 family members from other species were aligned to putative CDK1-3 family members (A) and other CDK or CDK-related sequences (B) identified in *B. minutum*. Sixteen residues flanking the cyclin binding motif are shown. Conserved amino acids are marked by an asterisk in the bottom row.

**Figure 5:**
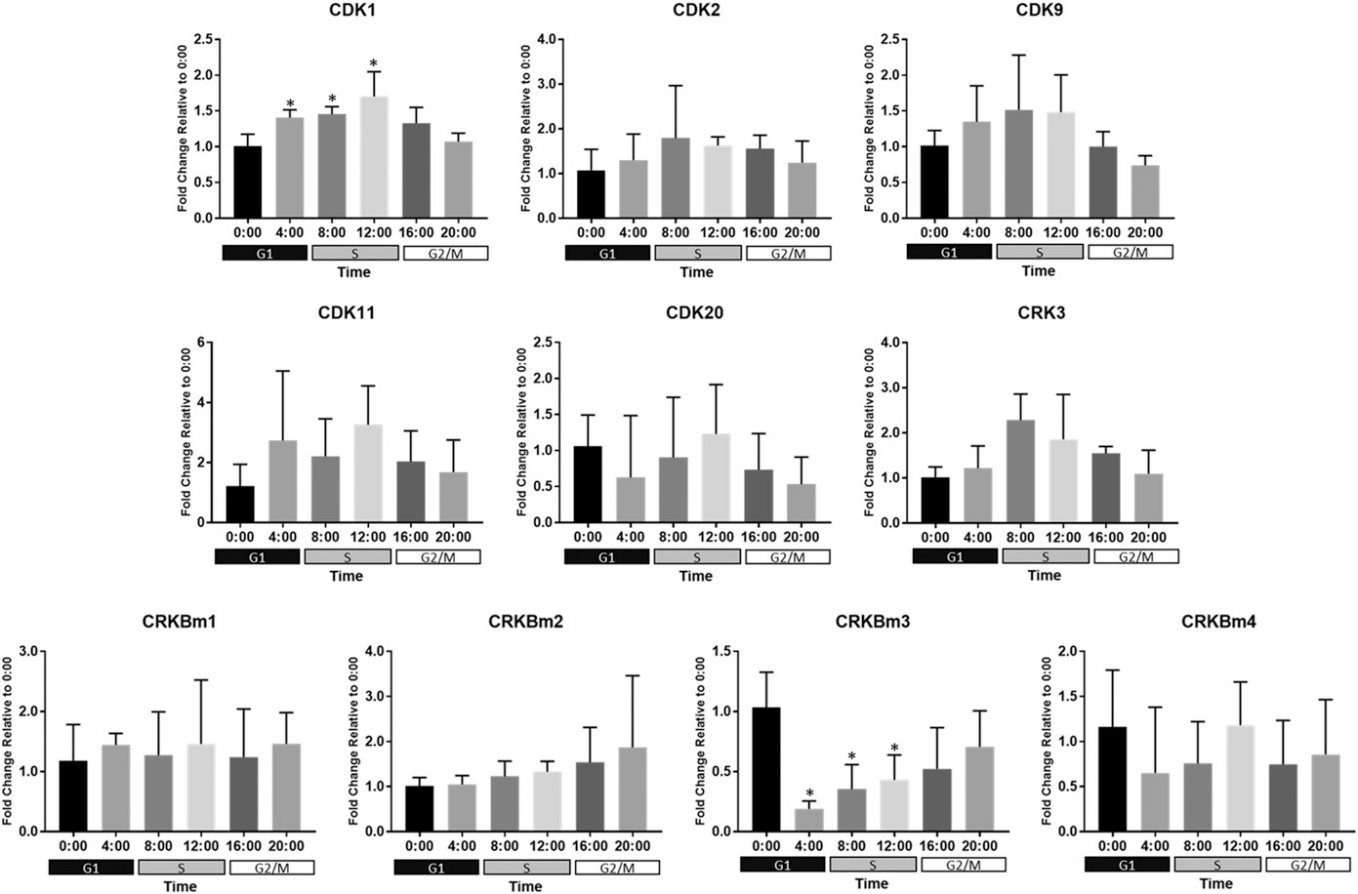
Expression of putative CDK and CDK-related genes in *B. minutum*. Messenger RNAs were isolated every 4 h from 3-4 cultures of free-living *B. minutum* maintained on a diurnal cycle. Following reverse transcription, qPCR was run with gene-specific primers. Data were analyzed and plotted as the average fold change relative to the 00:00 time-point ± SD. (*) indicates that expression at that time-point is significantly different than at 00:00 (one-way ANOVA followed by Dunnett’s test; *P* < 0.05). Shaded boxes below the x-axes indicate the predominant cell cycle phase at a given time-point.

Ten putative CDK and CDK-related sequences were identified in *B. minutum*; to group them within accepted subclasses (*e.g.*, CDK1, CDK20), phylogenetic trees were constructed with sequences from *H. sapiens*, *P. falciparum*, and *T. gondii*. *Plasmodium* and *Toxoplasma* are well-studied and share the superphylum Alveolata with Symbiodiniaceae, making them ideal for this analysis. Both neighbor joining and maximum likelihood methods yielded similar topologies (Figure 2 and Figure S4). Of the ten putative CDKs, two clustered with the CDK1-3 family. CDKs from this family are highly conserved (Figure 3) and all contain the highly conserved PSTAIRE motif, which is important for their association with the cyclins that are most responsible for cell cycle progression (Figure 4A). This motif is not conserved in the other CDK-related kinases that were identified in *B. minutum* (Figure 4B). While CDKs are regulated largely by post-translational modification and not at the transcriptional level, changes in CDK expression over the course of the cell cycle have been documented in other organisms (Bisova *et al.* 2005; Huysman *et al.* 2010). To determine if this is the case in *B. minutum*, we assessed expression of the CDKs over a 24 h period. Further supporting a role in cell cycle regulation, mRNA expression for the putative CDK1 is significantly higher from 04:00 to 12:00 than at onset of the light period (Figure 5). It is during this time period that we observe an increase in the percentage of S phase cells (starting after 04:00) and subsequently G2/M cells (starting after 08:00) (Figure 1). Based on all these data, we are confident in classifying these genes as cell cycle CDKs in *B. minutum*.

In addition to the canonical cell cycle CDKs, our phylogenetic analysis revealed putative CDK9, CDK11, and CDK20 homologs, a CDK-related kinase (CRK) that clusters with sequences from *Plasmodium* and *Toxoplasma*, as well as a group of four CRKs identified only in *B. minutum* (Figure 2 and Figure S4). The CDK9 family, including Ctk1p in *S. cerevisiae*, is involved in transcription initiation (Wallenfang and Seydoux 2002), and unsurprisingly, expression of *B. minutum* CDK9 does not depend on cell cycle phase (Figure 5). Similarly, expression of the CDK11 homolog followed no discernible pattern (Figure 5). In mammals, CDK11 has been shown to regulate RNA splicing in combination with cyclin L (Loyer *et al.* 2008); interestingly, three cyclin L homologs seem to be present in *B. minutum* (see below). As for the CDK20 homolog, no significant change in mRNA expression was observed over 24 h (Figure 5). Interestingly, in the green algae, *Chlamydomonas reinhardtii*, a CDK20 homolog is critical for assembly of flagella (Tam *et al.* 2007). Green algae are quite distinct from dinoflagellates, but a similar role for CDK20 may be worth investigating. Finally, it is unknown whether the cluster of four *B. minutum*-specific CDKs is unique to the species, the genus *Breviolum*, a larger group within the Symbiodiniaceae, or beyond. Until phylogenetic analysis of these genes across taxa is performed, we will refer to these as *B. minutum*-specific CDK-related kinases (CRKBm1-4). Of these, only CRKBm3 changed significantly over 24 h, with its peak expression during the dark period (Figure 5), suggesting that if it is involved in cell cycle regulation, it may have a role in preparing the cell for mitosis.

### Cyclins

**Figure 6:**
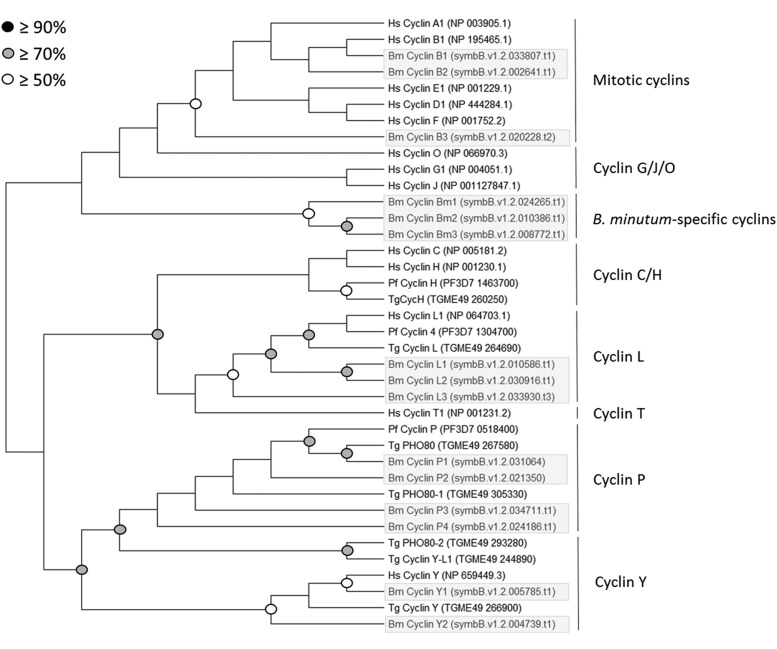
Phylogenetic analysis of putative cyclins in *B. minutum*. Protein sequences for the N-terminal domains (identified using PFAM) of 15 putative cyclins uncovered in the genome of B. *minutum* were aligned with representative cyclin sequences from other species. Using MEGA X, gaps were removed and phylogenetic trees were created. This tree was constructed from a MUSCLE alignment using neighbor joining with 1,000 replicates. Accession numbers or specific genome identifiers (for *B. minutum*) are indicated next to the sequence ID.

**Figure 7:**
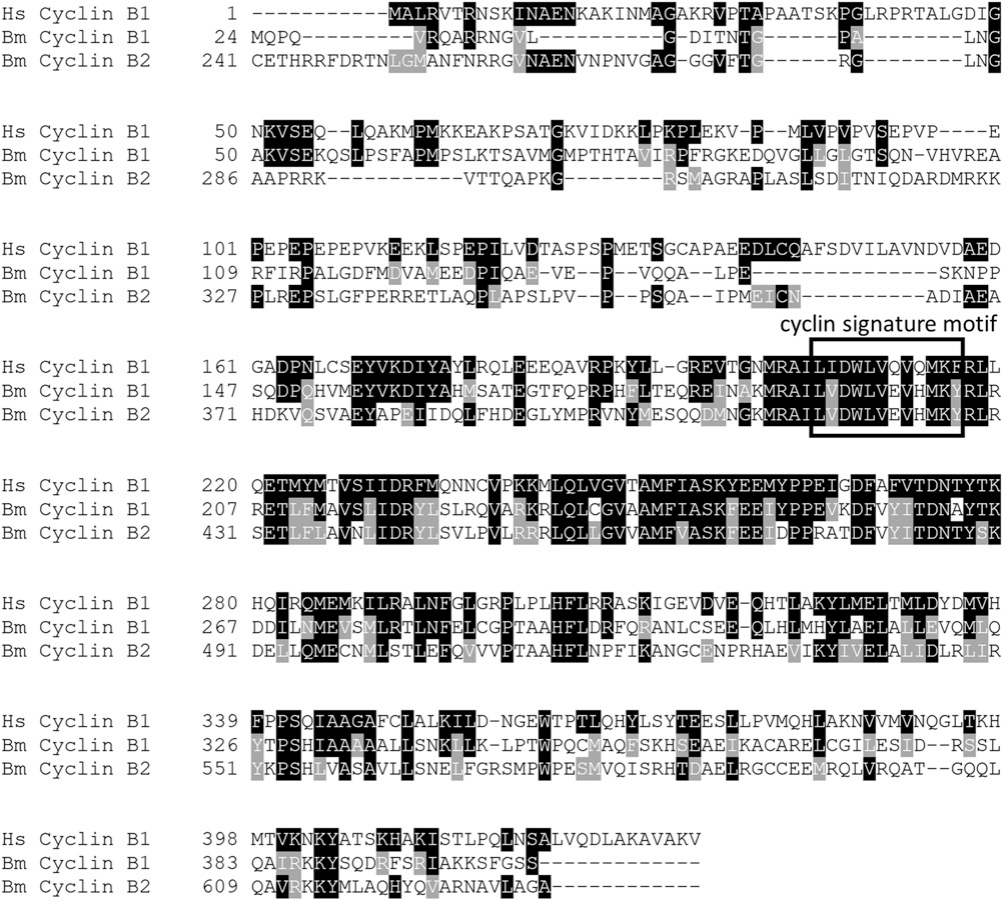
Alignment of putative *B. minutum* B-type cyclins with *H. sapiens* cyclin B1. Protein sequences for putative *B. minutum* cyclins B1 and B2 were aligned to *H. sapiens* cyclin B1 using Clustal Omega (Sievers *et al.* 2011) with default parameters. The BoxShade web server (expasy.org/resources) was used to shade the results of the alignment. Black boxes indicate an identical match to the consensus sequence (Hs Cyclin B1). Gray boxes indicate residues that are biochemically similar to the consensus sequence. The cyclin signature motif is outlined; the accepted consensus sequence for this motif in B-type cyclins is Lx[E/Q]VHxKF (Ma *et al.* 2013).

**Figure 8:**
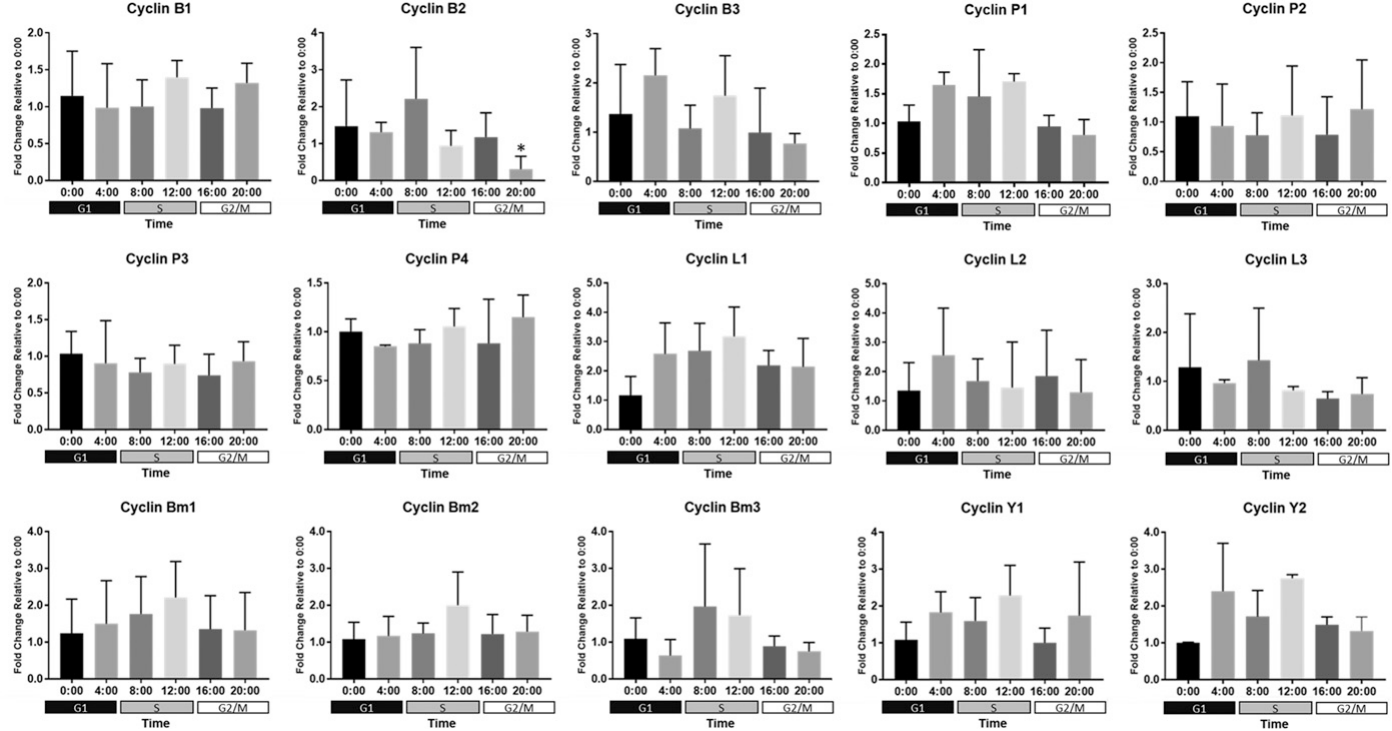
Expression of putative cyclin genes in *B. minutum*. Messenger RNAs were isolated every 4 h from 3-4 cultures of free-living *B. minutum* maintained on a diurnal cycle Following reverse transcription, qPCR was run with gene-specific primers. Data were analyzed and plotted as the average fold change relative to the 00:00 time-point ± SD. (*) indicates that expression at that time-point is significantly different than at 00:00 (one-way ANOVA followed by Dunnett’s test; *P* < 0.05). Shaded boxes below the x-axes indicate the predominant cell cycle phase at a given time-point.

Fifteen putative cyclins were identified in *B. minutum*. From the N-terminal domain that is present in all cyclins, phylogenetic trees were constructed. Both neighbor joining and maximum likelihood methods yielded similar topologies (Figure 6 and Figure S5). The branch support for most nodes is weak, especially for the maximum likelihood tree. This is not surprising due to the large evolutionary distance between these taxa and the fact that the cyclins belong to such a diverse gene family. Other studies—even those across a larger set of diverse taxa—also struggle with bootstrap replication of cyclin phylogenies (Cao *et al.* 2014). Still, several groups (*e.g.*, the mitotic cyclins and P-type cyclins) do stand out. Based on our phylogenetic analysis, the *B. minutum* cyclins were grouped within accepted subclasses (*e.g.*, cyclin A, cyclin B). This grouping revealed two sequences similar to known cyclin A and B family members (referred to as cyclins B1 and B2) and another (cyclin B3) that is generally similar to the family of mitotic cyclins, but not readily identifiable with a specific group. The cyclin B name was chosen because this family is ancestral to all mitotic cyclins and the cyclin signature motif in *B. minutum* cyclins B1 and B2 (LVEVHMKY) is more similar to the B-type motif (Lx[E/Q]VHxKF) than the A-type motif (LxEVx[D/E]EY) (Figure 7, Ma *et al.* 2013). The expression of cyclin B2 seems to peak around 08:00 and is significantly reduced by 20:00 (Figure 8). The percentage of S phase cells is beginning to increase by 08:00 (Figure 1). In canonical models, A-type cyclins are active in S phase and are necessary to initiate DNA replication (Girard *et al.* 1991), while B-type cyclins are the cyclin that is necessary for proper spindle assembly during mitosis (Michel *et al.* 2004). Since A-type cyclins seems to be absent in the lineage leading to Alveolates (Cao *et al.* 2014), it is possible that one or more of these putative B-type cyclins functions in S phase in *B. minutum*.

Interestingly, no A-type or B-type cyclins have been identified in *P. falciparum* or *T. gondii*.

In addition to the three putative B-type cyclins, other putative cyclins we identified include four P-type, three L-type, two Y-type, and three that do not fit phylogenetically into the well-established families. The four P-type cyclins are particularly interesting because cyclins belonging to this family are thought to be involved in phosphate signaling (Torres Acosta *et al.* 2004). Such a link connecting phosphate signaling to cell cycle regulation would be unsurprising in *Breviolum* since concurrent nitrogen and phosphate limitation can induce G1 arrest in these algae (Smith and Muscatine 1999). Under the conditions we tested, the P-type cyclins have relatively steady expression across 24 h (Figure 8). Since inorganic phosphate is abundant in f/2 media, consistent expression of P-type cyclins is unsurprising.

L-type cyclins pair with CDK11 and are important for RNA splicing (Dickinson *et al.* 2002; Loyer *et al.* 2008). As with the P-type cyclins, expression of the three cyclin L homologs in *B. minutum* is steady across 24 h (Figure 8).

The Y-type cyclins are highly conserved members of the cyclin family that have been studied exclusively in multicellular organisms. They appear to function as sensors linking extracellular signals with core cell cycle machinery (Liu *et al.* 2010). In *B. minutum*, we have identified two Y-type cyclins that are expressed consistently across 24 h (Figure 8).

Finally, three cyclin-like sequences we identified do not fit phylogenetically into the well-established families; however, a variety of cyclin sequences were returned as top hits, when these sequences were checked against the UniProtKB database using the HMMER web server, leading us to classify these as *B. minutum*-specific cyclins. As with the *B. minutum*-specific CDKs, it is unknown whether these cyclins are unique to the species, the genus *Breviolum*, to a larger group within the Symbiodiniaceae, or beyond.

It is somewhat surprising that analysis of gene expression revealed minimal changes in expression of *B. minutum* cyclins over 24 h. Unlike CDKs, many cyclins are known to change dramatically in abundance over the course of the cell cycle, and while post-translational regulation is important (*e.g.*, ubiquitination and proteasomal breakdown (Holloway *et al.* 1993)), transcription definitely plays a role (Fung and Poon 2005). It is possible that we don’t see significant changes in cyclin gene expression simply because our population of cells is not completely synchronized (*e.g.*, only about 30% of cells are in S phase from 08:00 to 12:00). Additionally, recent work suggests a minimal role for gene regulation in Symbiodiniaceae (Leggat *et al.* 2011; Barshis *et al.* 2014). This finding could explain the surprising lack of specific transcription factors uncovered in the genomes of this family (Bayer *et al.* 2012; Shoguchi *et al.* 2013). Thus, transcriptional control of cyclins may not be important for cell cycle regulation in this organism. Importantly, microRNAs and RNAi machinery have been uncovered in Symbiodiniaceae, indicating a possible role for post-transcriptional control in modulating these cell cycle regulators (Baumgarten *et al.* 2013).

Oddly, no cyclin D homolog was uncovered. This family of cyclins is involved in bringing cells out of quiescence and driving them through G1 (Resnitzky *et al.* 1994). Cyclin D appears to have arisen early in the eukaryotes, so the lack of a homolog in *B. minutum* likely reflects a deletion somewhere in its lineage (Cao *et al.* 2014). Similarly, no cyclin E homolog was detected in *B. minutum*. Initial work suggested that cyclin E arose in animals (Ma *et al.* 2013), however, analysis of multiple unicellular choanoflagellates uncovered a cyclin E ortholog (Cao *et al.* 2014). Cyclin E seems to have arisen in the lineage leading to choanoflagellates and animals after it split from the lineage that led to dinoflagellates.

### CDK activators and inhibitors

In addition to the presence or absence of a cyclin partner, CDKs are regulated by numerous other modulators. The CAKs and Cdc25 phosphatases, for instance, work in concert to add activating phosphorylations and remove inhibitory phosphorylations from CDKs (Kaldis 1999; Perry and Kornbluth 2007). In contrast, members of the WEE1/MYT1 kinase family phosphorylate CDKs in a way that renders them inactive (Perry and Kornbluth 2007). Further regulating CDK activity, the CDK-interacting protein CKS1 functions as a docking protein that allows CDKs to interact with their substrates (Tang and Reed 1993). Finally, the CKIs can block CDK activity and initiate cell cycle arrest in response to internal or external stimuli (El-Deiry *et al.* 1994; Polyak *et al.* 1994).

**Figure 9:**
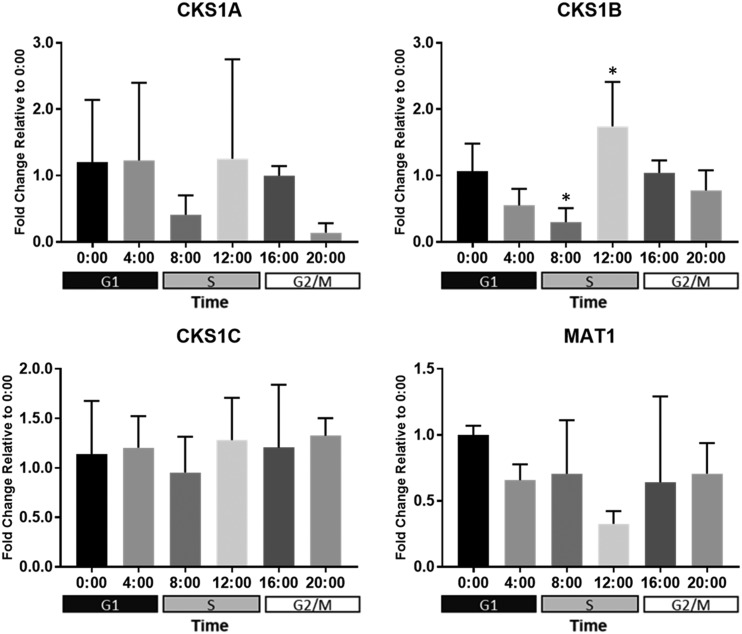
Expression of putative cyclin/CDK interacting partners in *B. minutum*. Messenger RNAs were isolated every 4 h from 3-4 cultures of free-living *B. minutum* maintained on a diurnal cycle Following reverse transcription, qPCR was run with gene-specific primers. Data were analyzed and plotted as the average fold change relative to the 00:00 time-point ± SD. (*) indicates that expression at that time-point is significantly different than at 00:00 (one-way ANOVA followed by Dunnett’s test; *P* < 0.05). Shaded boxes below the x-axes indicate the predominant cell cycle phase at a given time-point.

From this list of CDK activators and inhibitors, only a truncated sequence for a CAK subunit and three putative CKS1 homologs were identified in *B. minutum*. The CAK subunit was identified as MAT1—one part of a trimeric CAK complex (Kaldis 1999). Its expression does not change significantly over 24 h (Figure 9). Canonically, MAT1 functions as an assembly factor, forming a CAK with cyclin H and a D-type CDK (CDK7 in *H. sapiens*). Since we identified no cyclin H or CDKD homologs in *B. minutum*, it is possible that this partial MAT1 plays another role, perhaps independent of the cell cycle. The evolutionary history of this sequence might hold clues to its function. As for the three CKS1 homologs, only CKS1B expression changed significantly, reaching its highest level just before darkness (12:00; Figure 9). Similarly, CDK1 expression peaks around this time (Figure 5), perhaps hinting that these genes function as partners. In budding yeast, CKS1 is necessary for activation of the G1 cyclin/CDK pair (Reynard *et al.* 2000). It’s interesting, however, that the expression of CKS1B is at its lowest at 08:00 but its highest at 12:00. Since the percentage of S phase cells is already increasing by 08:00, this suggests that CKS1B is not involved in the G1/S transition but, instead, plays a role later—perhaps getting cells into G2/M.

No members of the WEE1/MYT1 family of CDK-inhibiting kinases were identified; thus, it is not surprising that their antagonists—the Cdc25 phosphatases—were also absent. Finally, no CKIs—small CDK inhibitors such as the mammalian proteins p21^Cip1/Waf1^ and p27^Kip1^—were uncovered in the *B. minutum* genome. It is possible that one or more CKIs are present but are simply undetectable using our methods, which rely on a large degree of sequence similarity that is uncharacteristic of the CKIs.

## Conclusion

Unsurprisingly, our analysis of key cell cycle regulators in the genome of *B. minutum* has revealed that control of mitotic cell divisions in these dinoflagellates shares some features with other eukaryotes. For instance, the two cell cycle CDKs that were identified both contain a highly conserved PSTAIRE motif, suggesting that they regulate cell cycle progression directly. Further, expression of CDK1 and cyclin B2 are both highest as cells are transitioning from G1 to S phase, suggesting these proteins might form a cyclin/CDK pair that may be involved in driving cells through S phase, much like cyclin A/CDK2 in mammals. Regulation of S phase entry is particularly interesting because eukaryotic cells seem to commit to division at a point in G1 or at the G1/S transition. As a general rule, the cell cycle is only sensitive to external factors prior to this commitment point (Pardee 1974; Hartwell *et al.* 1974). Thus, if a Cnidarian host is regulating cell divisions in its endosymbiont, it is likely that it does so by controlling the G1/S transition that may be regulated by the *B. minutum* cyclin B2/CDK1 pair. Knowing the identity of these cell cycle regulators is the first step in designing experiments to determine the molecular events that are responsible for maintaining Cnidarian control over symbiont life cycles.
